# Neonatal endotracheal intubation: development of a comprehensive educational program for procedural skills

**DOI:** 10.1038/s41390-025-04217-4

**Published:** 2025-06-17

**Authors:** Sachin Agrawal, Abhijeet Rakshasbhuvankar, Chandra Rath, Janie A. Brown, Peter M. McEvoy, Shripada Rao, Sanjay Patole

**Affiliations:** 1 School of Medicine, University of Western Australia, Crawley, WA Australia; 2 King Edward Memorial Hospital for Women, Subiaco, WA Australia; 3 Perth Children’s Hospital, Nedlands, WA Australia; 4 School of Nursing, Curtin University, Bentley, WA Australia; 5 The Western Australian Group for Evidence Informed Health Practice: A JBI Centre of Excellence, Bentley, WA Australia; 6 enAble Institute, Curtin University, Bentley, WA Australia; 7 School of Population Health, Curtin University, Bentley, WA Australia; 8 Centre for Clinical Interventions, North Metropolitan Area Health Service, Northbridge, WA Australia

## Abstract

**Background:**

Acquiring skills for neonatal endotracheal intubation (ETI) has become difficult for trainees due to decreased opportunities following the increased use of non-invasive respiratory support. Recent studies report very low rates of successful first-attempt ETI in neonates despite current educational strategies. Hence, novel approaches for coaching the trainees to become procedurally competent are urgently required.

**Methods:**

A comprehensive literature review of PubMed, Embase, Emcare, CINAHL, and Cochrane Library databases was conducted in January 2025 to identify evidence-based strategies to improve neonatal ETI skills. A multidisciplinary approach was taken for integrating stress-management skills in the educational program.

**Results:**

Video-laryngoscopy aided coaching, Just-in-time training, frequent practice on mannequins, rapid cycle deliberate practice, mental skills training, and deep-slow breathing were identified as appropriate strategies for inclusion in the educational program. Based on the principles of micro-credentialing, a comprehensive educational program was developed.

**Conclusions:**

Our innovative educational program focuses on enhancing procedural skills for neonatal ETIs and reducing acute stress response in trainees while performing the procedure. Well-designed studies are needed to evaluate the impact of the program on patient outcomes and trainee wellbeing. Whilst primarily aimed at trainees, even senior fellows, consultants and nursing staff can use it to maintain their skills.

**Impact:**

- Our program facilitates coaching of trainees in neonatal endotracheal intubation skills.
- It provides feasible solutions to attenuate stress while performing the procedure.
- Whilst primarily aimed at trainees, even senior clinicians can use it.
- The program has the potential to improve patient safety and trainee wellbeing.
- It can be adopted for other high-acuity low-occurrence (HALO) clinical procedures.

## Background

With the increasing use of non-invasive modes of ventilation and increased number of trainees, opportunities for endotracheal intubation (ETI) in neonates have decreased significantly in recent years.^1,2^ ETI is currently considered a High Acuity and Low Occurrence (HALO) procedure.^3^

A large international study found that 1697 (22%) out of 6620 neonatal ETIs met the definition of difficult intubation (≥three attempts).^4^ Difficult intubations were more common in very preterm (<32 w gestation) and very low birth weight (<1500 g) infants. A study conducted in the United Kingdom (UK) found that the first attempt success rate was only 53% and 23% required ≥3 attempts.^5^ Similar findings were obtained in the national survey of general paediatric/neonatal trainees or advanced nurse practitioners in the UK, with only 18% of the respondents feeling completely confident about intubation.^6^ The issue is even more serious in non-tertiary hospitals where the attending paediatricians have limited opportunities but are expected to intubate a critically ill neonate. In two surveys of non-tertiary Scottish neonatologists, only 57% reported feeling confident, falling to 37% for infants under 28 weeks’ gestation. Most (63%) had experienced difficult intubation, and many considered it to be a traumatic event.^7^

Failure to intubate or taking multiple attempts can have devastating consequences for the infant including pneumothorax, airway injury, severe oxygen desaturation, bradycardia, brain injury and mortality.^8^

Inexperience, a large audience during intubation, parental presence, infant instability, and delivery room intubation, are known to increase stress among the intubators.^9^ Team stress is known to be associated with adverse events during neonatal tracheal intubations.^10^ The presence of senior members has the potential to decrease stress levels. However, as noted by Edwards et al.^7^ even senior members are finding it challenging to maintain procedural competency, and hence, their presence is unlikely to reduce stress levels among trainee doctors during ETIs.

Trainees and clinicians who are unsuccessful in intubating critically ill neonates are at risk of burnout and not meeting the competency requirements of professional colleges. It has significant consequences for the organisation in the context of reputation, accreditation, and litigations. Considering such disastrous consequences to all stakeholders, the importance of acquiring and maintaining ETI skills needs special emphasis.

Effective communication, planning, situational awareness, and keeping a professional demeanour are critical for the success of a complex and time-critical procedure such as ETI. Application of such skills is possible if clinicians can control their own acute stress response, the so-called “amygdala hijack”.^11–13^ The psychological stress associated with ETIs could adversely affect an individual’s capacity to apply learned skills during other real-life stressful situations.^13^ Therefore, a feasible and effective approach to attenuate acute psychological stress while performing neonatal ETI is highly desirable.

Traditional neonatal resuscitation simulation workshops have been around for decades.^14^ However their scope is broad, and they are not focused specifically on enhancing ETI skills. Moreover, one attends such workshops infrequently and the skills gained decay quickly.^15,16^ Strategies to minimise psychological stress receive little attention during such workshops. Hence, newer coaching strategies are urgently needed to acquire and maintain neonatal ETI skills as well as reduce psychological stress.

We aimed to develop a novel and comprehensive educational program for (1) acquiring and maintaining the skills for neonatal ETI, (2) attenuating the acute mental stress response during the peri-procedural period, and (3) selecting a format appropriate for training in and monitoring competency in the organisational context.

## Methods

(1) We performed a comprehensive literature review to identify evidence-based strategies to acquire and maintain neonatal ETI skills, (2) a multi-disciplinary team including clinical psychology, nursing and medical clinicians, and clinical academics identified strategies to reduce stress while performing ETI, and (3) had group discussions to select feasible and evidence-based strategies for inclusion in the educational program.

A comprehensive literature review of PubMed, Embase, Emcare, CINAHL, and Cochrane Library databases was conducted in January 2025. The search terms used for individual databases are given in Supplement S1. The following types of studies were included: randomised controlled trials (RCTs), observational studies and systematic reviews where trainees were involved, and outcomes were on real infants (not just mannequins/simulators).

The following types of studies were excluded: Studies in which trainees were not involved, studies focussing on difficult airways, fibreoptic bronchoscopes, simple checklists, studies focussing on methods confirming the position of the endotracheal tube, studies focussing on practising intubation on infant cadavers and animals, studies involving children more than one year of age, laryngeal mask airway and other airway adjuncts and studies focussing on simulators/mannequins without assessing the efficacy in human infants.

## Results

### Literature review

The database search yielded 1701 articles. After removing 439 duplicates and going through the titles and abstracts of the remaining 1262 articles, 25 appeared relevant. We reviewed their full texts and identified 10 studies describing four educational strategies pertinent to our aim: Video-laryngoscopy assisted coaching,^17–22^ Just-in-Time training (JiTT),^23,24^ frequent practice on mannequins,^25^ and Rapid Cycle Deliberate Practice (RCDP).^26^ The flow diagram of the study selection process is given in Supplementary Fig. S1.

### Video-laryngoscope-assisted coaching

Video-laryngoscopy (VL) enables on-screen visualization of laryngeal structures through high-resolution cameras and video systems.^27,28^ It offers advantages for coaching on the mannequins as well as while performing intubation on real babies, by providing a better view of the vocal cords, and by being less traumatic.

Many RCTs and systematic reviews have reported that VL improves first-attempt success rates of neonatal ETIs.^29,30^ Hence, we specifically looked for evidence of the utility of VL in teaching neonatal ETI skills to trainees. Since the supervisors can see the anatomy on the VL screen when the trainees are attempting ETI, it makes easy to correct the technique in real-time.

O’Shea et al.^17^ randomised neonatal intubations done by doctors with six months’ tertiary neonatal experience to having the VL screen visible to the instructor or covered. Two hundred six first-attempt intubations were analysed. The success rate when the instructor was able to view the VL screen was 66% (69/104) compared with 41% (42/102) when the screen was covered (Odds Ratio 2.81, 95% CI 1.54–5.17). They concluded that intubation success rates of inexperienced trainees improved when the instructor was able to share their view on a VL screen.

Volz et al.^19^ randomised 48 first and second-year residents to perform neonatal intubations using VL or direct laryngoscopy (DL). Of the 101 intubations analysed, the success rate was greater in the VL vs. DL group (57% vs. 33%). First-year residents and residents intubating their first patient had higher success rates in the VL vs. DL group (58% vs. 23% and 50% vs. 17%, respectively). They concluded that coaching using VL improved neonatal intubation success rates.

Saran et al.^18^ in a randomised cross-over study of 24 trainees performing ETI on 144 infants aged 1 day to 6 months found the first attempt success rate to be higher with VL-assisted feedback compared to conventional feedback (83.3% vs. 44.4%). The time to best glottic view (19.8 s vs. 26.8 s) and intubation (30 s vs. 41.7 s) were significantly shorter in the VL-assisted group. They concluded that VL-assisted verbal feedback resulted in a high success rate and reduced complications.

In the RCT by Moussa et al.^20^ 34 paediatric residents performed 213 intubations by using either VL or DL. The success rate was higher (75.2% vs 63.4%, *P* = 0.03), but time to intubation was longer in the VL group (57 vs 47 s, *P* = 0.008). They concluded that VL is a promising tool for teaching neonatal ETI.

In the RCT by Yankowski et al.^22^ 49 neonatal learners with <1-year neonatal intubation experience were randomized to either VL/DL (*n* = 25) or standard DL (*N* = 24) intubation curriculums. Compared to the VL/DL curriculum, participants in the standard DL curriculum were more successful in the clinical intubation setting (70% vs 33%, *p* = 0.048). They concluded that a VL-based educational curriculum could negatively impact early learners’ intubation skills.

The systematic review by MacKinnon et al.^31^ concluded that VL, combined with real-time supervisor feedback, is an effective tool for acquiring neonatal intubation skills among trainees.

Overall, the evidence indicates that VL is a useful tool to teach ETI skills to neonatal trainees on the mannequins as well as while performing intubations on real babies.

### Just-in-time training

Just-in-time training (JiTT) involves training in the form simulations with mannequins to strengthen previously learned skills immediately before performing the clinical procedure.^23,24^ In the context of neonatal endotracheal intubations, it involves practising the intubation on a mannequin immediately prior to performing it on the real baby. Simply watching the video of intubation cannot be counted as JiTT; actual hands-on practice is essential as it improves immediate muscle memory.

JiTT has been used to improve procedural competency and patient outcomes in surgical and anaesthetic fields. Performing warm-up (i.e., JiTT) laparoscopy on mannequins just before doing it on real patients improves the surgeon’s technical skills and patient outcomes.^32^ Similarly, performing warm-up flexible bronchoscopy on mannequins just before the actual procedure on real patients can improve the success rates of anaesthetists.^33^ A recent systematic review that included the adult/paediatric/neonatal population concluded that JiTT is useful for improving learning and performance outcomes.^34^

In the recent RCT by Flynn et al.^24^ from Boston Children’s Hospital, 153 trainees (paediatric anaesthesia fellows, residents, and nurse anaesthetist students) were randomized to receive either JiTT, consisting of expert coaching on a mannequin within one hour before intubating real infants, or standard intraoperative instruction. They found that the JiTT group had a higher first-attempt success rate (212/213; 91.4%) compared to the control group (231/283; 81.6%); adjusted odds ratio 2.42 (CI: 1.45–4.04). Secondary outcomes also favoured the JiTT group, showing decreased cognitive load and improved competency. They concluded that JiTT enhances infant intubation success and could improve patient safety in other high-stakes procedures. While positively commenting on the trial design and its results, experts have suggested that JiTT is a type of point-of-care training and continuous professional development with the potential to improve patient outcomes and protect the mental health of its users.^35^

In a multicentre RCT, Gizicki et al.^23^ assessed if simulation-based JiTT (short video and simulation) is superior to video training (5-min video) in acquiring neonatal ETI skills. Sixty-five residents performed 139 ETIs. The overall success rate was similar for both groups (67% vs 70%, *P* = 0.71). However, the first-attempt success rate was higher for the JiTT group (54% vs 41%, *P* = 0.035). The mean (SD) duration of attempts was shorter [35 (9) vs 62 (9) seconds, *P* = 0.048)] in the JiTT group. They concluded that simulation-based JiTT can become an educational adjunct to neonatal ETI training for residents.

These data support the usefulness of JiTT as an effective adjunct strategy to enhance procedural competency in neonatal ETI.

### Frequent practice on simulator mannequins followed by performing ETI on real infants

JiTT will not mitigate the risk in situations requiring emergency intubations. To overcome this limitation, it is important for clinicians to practice frequently on the mannequin (rolling- refreshers). The situation is akin to the need for regular military training so that the team is ready for acute emergencies.^36^

In a prospective study, Dalrymple et al.^25^ aimed to increase the first-pass intubation rate by introducing daily intubation simulation at their mixed neonatal and paediatric retrieval service. In total, 16 medical staff performed simulated intubation at the commencement of their retrieval shift with a retrieval nurse. Seventy patients required intubation by the retrieval team during the intervention period. First-pass intubation rates were higher during the intervention period compared with a historical cohort despite fewer intubations being performed overall. First-pass intubation rates improved from 59% to 78% in neonatal patients (*P* = 0.032), 58% to 65% in paediatric patients (*P* = 0.68) and from 58% to 74% overall (*P* = 0.043). They concluded that simulation is a useful adjunct to support neonatal and paediatric intubation training in the current environment of reduced intubation frequency.

### Rapid cycle deliberate practice

Adequate supervision of trainees is essential while they perform simulations to ensure their technique is correct. This can be achieved through RCDP, which is an instructional method with rapid cycling between deliberate practice and feedback until mastery of the skill is achieved.^37^ This approach helps create muscle memory for the “right way” of performing ETI. Since the feedback is in real-time and frequent, it could be perceived as confrontational by young trainees, and hence, adequate safeguards should be in place while implementing it.

A recent study found that resident medical officers in the RCDP group performed better than the control group for overall neonatal resuscitation performance (65% vs 87%, *p* = 0.004), administering positive-pressure ventilation (63% vs 88%, *p* = 0.006), and validated behaviour skills (1.4 vs 2.0, *p* = 0.019). Residents in the RCDP group reported greater confidence in the resuscitation of extremely low birth weight infants.^26^ Similar findings were noted in another study among interns.^38^ and nurses.^39^ A systematic review conducted on behalf of the International Liaison Committee on Resuscitation task force found that RCDP resulted in a significantly shorter time to defibrillation and time to administration of epinephrine than controls. They concluded that it is reasonable to include RCDP as an instructional design feature of basic and advanced life support training.^37^

### Strategies to attenuate trainee’s stress response during ETI

Attenuating trainees’ acute stress response is crucial while they are performing time-critical procedures like ETI. Two evidence-based, simple, and effective strategies that appear beneficial in this context are deep slow breathing (DSB).^40–42^ and mental skills training (MST).^43,44^

Acute stress response involves hyperventilation leading to hypocarbia and respiratory alkalosis with symptoms such as anxiety, light-headedness/dizziness, dry mouth, tingling, nausea, and shortness of breath.^45^ The adverse effects of hyperventilation can be easily reversed by reducing respiration rate through DSB, which will restore blood pH and reduce uncomfortable physical sensations.^38,40,41^ A low inhale/exhale ratio increases vagal activity, resulting in a relaxed state of mind. Various studies have shown that DSB is a simple and effective technique for reducing acute stress responses.^40–42,45,46^ A simple method for DSB is the *“4-7-8 breathing”*, which can be practised anywhere and at any time.^47^ The details are covered in the supplemental file (Supplement S5).

MST is a rehearsal involving mental imagery without physically performing the task.^43,44,48^ It is a well-established tool in aviation, elite sports, and arts. It incorporates contextual and multi-sensory components in a variety of ‘real-world’ scenarios and is known to improve performance.^49,50^ Many systematic reviews have shown the benefits of MST in learning procedural skills and reducing stress and burnout.^43,44,49–54^ The method of MST in our program is to mentally go through the steps of intubation anywhere and anytime, including just before practising on mannequins and real-life intubation (Supplement S6).

### Development of the program and resources

Based on the review of literature and group discussion, video-laryngoscopy assisted coaching, JiTT, frequent practice on mannequins, RCDP, MST, and DSB were identified as strategies appropriate for inclusion in the educational program.

**Fig. 1:**
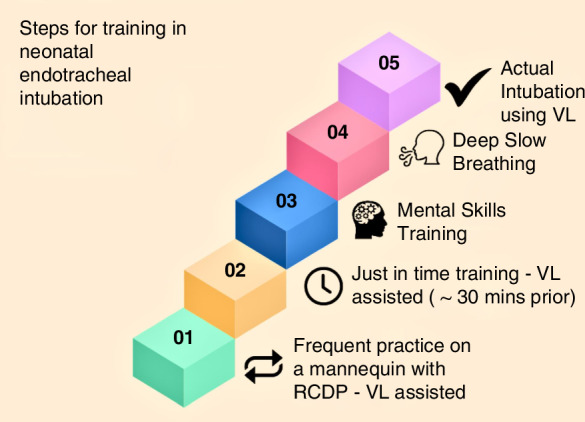
Mind map: steps for training in neonatal endotracheal intubation.

Based on these identified strategies, we developed a comprehensive educational program for acquiring and maintaining ETI skills (Fig. 1 and Supplements S3–S7) after consulting an expert in micro-credentials.

Micro-credentialing, the use of short, focused credentials that provide in-demand skills, was considered a suitable teaching format for implementing the educational program. We identified a wide range of studies supporting micro-credentials as a proper format for training in procedural skills and monitoring skill competency.^55–68^ Employers use micro-credentials to encourage continued professional development and address emerging workplace issues.^69^

We included YouTube educational videos (with permission) suitable for neonatal ETI training and prepared an ETI instruction card, a performance checklist, and instructions for DSB and MST (Supplements S3–S7). Finally, we developed a framework for implementing and assessing the program as a quality improvement project (Supplement S8). Our program includes a standardised and structured approach to learning, instruction, supervision, practice, assessment, and credentialing (Fig. 1). It is based on the recommended Australian National Framework for developing micro-credentials (Supplement S2).

## Discussion

We have developed a comprehensive and innovative educational program to improve the acquisition and maintenance of skills essential for a HALO procedure such as neonatal ETI among trainees. In addition to VL-assisted intubation, JiTT, frequent simulation practice and RCDP, the comprehensive program includes simple and effective psychological strategies that trainees can easily use to reduce their acute stress response during intubation.^34,37,39,67,69^ Importantly, our program is based on the national framework for micro-credentials, an emerging educational tool.^70^ Micro-credentials provide the advantage of spaced compared with massed learning where a large amount of information is covered in a single session.^71^

A recent scoping review identified video laryngoscopy, virtual reality training, simulation-based training and supervised clinical practice as potentially useful strategies for training in neonatal intubations.^72^ However, they did not develop the actual training program. Moreover, they did not cover strategies to mitigate psychological stress while performing neonatal ETIs. Their outcome settings were both simulators and real infants, while our outcome setting was real infants.

Although the individual components of our program have been assessed in clinical studies, the effectiveness of the entire program needs to be measured with learning and clinical outcomes using either a randomised trial or a quality improvement design (Supplement S8). It could be used by any organisation and modified according to local needs and resources. The effectiveness of the program can be assessed using pre-defined deliverables and outcomes (Supplement S8). The educational program could also be adapted for training in other HALO clinical procedural skills, such as the insertion of chest drains, nasotracheal intubations, intraosseous lines and others.

In summary, we have developed an evidence-based, comprehensive educational program to assist in the acquisition of the skills for ETI in neonates and prevent skill decay. Whilst meant for trainees, it can be used by senior staff members (i.e. consultants), nurses and nurse practitioners and other healthcare workers who are required to perform neonatal intubations.

## Supplementary information

Supplementary Information

## Data Availability

All data generated or analysed during this study are included in this published article [and its supplementary information files].

## References

[1] O’Donnell, C. P. F. Intubation difficulty in neonatology: are you experienced?. *Arch. Dis. Child Fetal Neonatal Ed.* **104**, F458–f460 (2019). 30796061 10.1136/archdischild-2018-316711

[2] Downes, K. J., Narendran, V., Meinzen-Derr, J., McClanahan, S. & Akinbi, H. T. The lost art of intubation: assessing opportunities for residents to perform neonatal intubation. *J. Perinatol.* **32**, 927–932 (2012). 22382858 10.1038/jp.2012.17

[3] Hack, K. E., Levy, M. J., Garfinkel, E. & Margolis, A. M. Establishing consensus-based high-acuity low-occurrence skills for ems physicians: a pilot survey of ems fellowship faculty. *AEM Educ. Train.* **6**, e10828 (2022). 36562031 10.1002/aet2.10828 PMC9763967

[4] Singh, N. et al. Impact of multiple intubation attempts on adverse tracheal intubation associated events in neonates: a report from the Near4neos. *J. Perinatol.* **42**, 1221–1227 (2022). 35982243 10.1038/s41372-022-01484-5

[5] Edwards, G. et al. Neonatal intubation success rates: four Uk units. *Arch. Dis. Child Fetal Neonatal Ed.* **105**, 684 (2020). 32414816 10.1136/archdischild-2020-319111

[6] Belkhatir, K., Scrivens, A., O’Shea, J. E. & Roehr, C. C. Experience and training in endotracheal intubation and laryngeal mask airway use in neonates: results of a national survey. *Arch. Dis. Child Fetal Neonatal Ed.* **106**, 223–224 (2021). 32571833 10.1136/archdischild-2020-319118

[7] Edwards, G., Van Der Heide, P. & O’Shea, J. E. Experience of endotracheal intubation and supraglottic airway insertion by consultant paediatricians in non-tertiary neonatal units: a scotland-wide survey. *Arch. Dis. Child Fetal Neonatal Ed.* **110**, 111–112 (2024). 38937075 10.1136/archdischild-2024-327257

[8] Sawyer, T. et al. Incidence, impact and indicators of difficult intubations in the neonatal intensive care unit: a report from the national emergency airway registry for neonates. *Arch. Dis. Child Fetal Neonatal Ed.* **104**, F461–f466 (2019). 30796059 10.1136/archdischild-2018-316336

[9] Bensouda, B. et al. Effect of an audience on trainee stress and performance during simulated neonatal intubation: a randomized crossover trial. *BMC Med. Educ.* **18**, 230 (2018). 30285715 10.1186/s12909-018-1338-4 PMC6171149

[10] Umoren, R. A. et al. Team stress and adverse events during neonatal tracheal intubations: a report from Near4neos. *Am. J. Perinatol.* **37**, 1417–1424 (2020). 31365934 10.1055/s-0039-1693698

[11] Shackman, A. J., Grogans, S. E. & Fox, A. S. Fear, anxiety and the functional architecture of the human central extended amygdala. *Nat. Rev. Neurosci.* **25**, 587–588 (2024). 38858579 10.1038/s41583-024-00832-y PMC11262955

[12] Kim, H. et al. Functional connectivity of the amygdala in relation to high stress and low mindfulness. *Neurosci. Lett.* **842**, 137985 (2024). 39255895 10.1016/j.neulet.2024.137985

[13] Almarzouki, A. F. Stress, working memory, and academic performance: a neuroscience perspective. *Stress* **27**, 2364333 (2024). 38910331 10.1080/10253890.2024.2364333

[14] Zaichkin, J., Kamath-Rayne, B. D. & Weiner, G. The Nrp 8th edition: innovation in education. *Adv. Neonatal Care* **21**, 322–332 (2021). 34397537 10.1097/ANC.0000000000000884

[15] Trevisanuto, D. et al. Knowledge gained by pediatric residents after neonatal resuscitation program courses. *Paediatr. Anaesth.* **15**, 944–947 (2005). 16238554 10.1111/j.1460-9592.2005.01589.x

[16] Branch, R. & Cole, K. Advanced airway management skill decay: a review of the literature. *Aana J.* **92**, 167–172 (2024). 38758710

[17] O’Shea, J. E. et al. Videolaryngoscopy to teach neonatal intubation: a randomized trial. *Pediatrics* **136**, 912–919 (2015). 26482669 10.1542/peds.2015-1028

[18] Saran, A., Dave, N. & Karnik, P. Efficacy and safety of videolaryngoscopy-guided verbal feedback to teach neonatal and infant intubation. A prospective randomised cross over study. *Indian J. Anaesth.* **63**, 791–796 (2019). 31649390 10.4103/ija.IJA_823_18 PMC6798639

[19] Volz, S., Stevens, T. P. & Dadiz, R. A randomized controlled trial: does coaching using video during direct laryngoscopy improve residents’ success in neonatal intubations?. *J. Perinatol.* **38**, 1074–1080 (2018). 29795452 10.1038/s41372-018-0134-7

[20] Moussa, A. et al. Neonatal endotracheal intubation learned with videolaryngoscope is maintained with classic laryngoscope: phase 2 of a crossover randomized trial. *Paediatrics Child Health* **20**, e33 (2015).26435676

[21] MacKinnon, J. & McCoy, C. Use of video laryngoscopy versus direct laryngoscopy as a teaching tool for neonatal intubation: a systematic review. *Can. J. Respir. Ther.* **59**, 111–116 (2023). 37056577 10.29390/cjrt-2022-056 PMC10089680

[22] Yankowski, C., Kovatis, K. Z. & Nassar, R. Impact of video laryngoscopy-enhanced advanced airway curriculum for learners in the neonatal intensive care unit. *J. Investig. Med.* **70**, 1129 (2022).

[23] Gizicki, E. et al. Just-in-time neonatal endotracheal intubation simulation training: a randomized controlled trial. *J. Pediatr.* **261**, 113576 (2023). 37353151 10.1016/j.jpeds.2023.113576

[24] Flynn, S. G. et al. Coaching inexperienced clinicians before a high stakes medical procedure: randomized clinical trial. *BMJ* **387**, e080924 (2024). 39681397 10.1136/bmj-2024-080924 PMC11648086

[25] Dalrymple, H. M. & Browning Carmo, K. Improving intubation success in pediatric and neonatal transport using simulation. *Pediatr. Emerg. Care* **38**, e426–e430 (2022). 33273427 10.1097/PEC.0000000000002315

[26] Hadfield, B. R., Sawyer, T., Moreira, A. G., Farner, R. & Vasquez, M. M. Rapid cycle deliberate practice improves resident performance during ELBW resuscitation. *J. Neonatal Perinat. Med.* **17**, 31–40 (2024). 10.3233/NPM-230102 38217617

[27] Coelho, L. P. & Couto, T. B. Can video laryngoscopy and supplemental oxygen redefine pediatric, infant and neonatal tracheal intubation standards?. *Transl. Pediatr.* **13**, 508–512 (2024). 38590366 10.21037/tp-23-530 PMC10998985

[28] Lingappan, K., Neveln, N., Arnold, J. L., Fernandes, C. J. & Pammi, M. Videolaryngoscopy versus direct laryngoscopy for tracheal intubation in neonates. *Cochrane Database Syst. Rev.* **5**, CD009975 (2023). 10.1002/14651858.CD009975.pub4 PMC10177149 37171122

[29] Kuitunen, I., Rasanen, K. & Huttunen, T. T. Video laryngoscopy in neonate and infant intubation-a systematic review and meta-analysis. *Eur. J. Pediatrics* **184**, 34 (2025). 10.1007/s00431-024-05839-2 PMC11579204 39565430

[30] Lingappan, K., Neveln, N., Arnold, J. L., Fernandes, C. J. & Pammi, M. Videolaryngoscopy versus direct laryngoscopy for tracheal intubation in neonates. *Cochrane Database Syst. Rev.* **5**, Cd009975 (2023). 37171122 10.1002/14651858.CD009975.pub4 PMC10177149

[31] MacKinnon, J. & McCoy, C. Use of video laryngoscopy as a teaching tool for neonatal intubation: a systematic review. *Can. J. Respir. Ther.* **59**, 111–116 (2023). 10.29390/cjrt-2022-056 PMC10089680 37056577

[32] Lee, J. Y. et al. Laparoscopic warm-up exercises improve performance of senior-level trainees during laparoscopic renal surgery. *J. Endourol.* **26**, 545–550 (2012). 22192095 10.1089/end.2011.0418 PMC3552180

[33] Samuelson, S. T. et al. Simulation as a set-up for technical proficiency: can a virtual warm-up improve live fibre-optic intubation?. *Br. J. Anaesth.* **116**, 398–404 (2016). 26821699 10.1093/bja/aev436

[34] Patocka, C. et al. The Impact of just-in-time simulation training for healthcare professionals on learning and performance outcomes: a systematic review. *Simul. Health.* **19**, S32–s40 (2024). 10.1097/SIH.0000000000000764 38240616

[35] Naylor, J. M. Just-in-time training could be just what the doctor ordered. *BMJ* **387**, q2747 (2024). 39681388 10.1136/bmj.q2747

[36] Mileder, L. P. Can learning from military history help us improving neonatal intubation success rates?. *Paediatr. Anaesth.* **34**, 1287–1288 (2024). 39254525 10.1111/pan.14996

[37] Abelairas-Gómez, C., Cortegiani, A., Sawyer, T., Greif, R. & Donoghue, A. Rapid cycle deliberate practice approach on resuscitation training: a systematic review. *Resusc Plus* **18**, 100648 (2024). 38757054 10.1016/j.resplu.2024.100648 PMC11096743

[38] Magee, M. J., Farkouh-Karoleski, C. & Rosen, T. S. Improvement of immediate performance in neonatal resuscitation through rapid cycle deliberate practice training. *J. Grad. Med Educ.* **10**, 192–197 (2018). 29686759 10.4300/JGME-D-17-00467.1 PMC5901799

[39] Yang, S. Y. & Oh, Y. H. Development and effectiveness of a rapid cycle deliberate practice neonatal resuscitation simulation program: a quasi-experimental study. *Healthcare* **12**, 104 (2024). 10.3390/healthcare12010104 PMC10779408 38201010

[40] Gerritsen, R. J. S. & Band, G. P. H. Breath of life: the respiratory vagal stimulation model of contemplative activity. *Front. Hum. Neurosci.* **12**, 397 (2018). 30356789 10.3389/fnhum.2018.00397 PMC6189422

[41] Magnon, V., Dutheil, F. & Vallet, G. T. Benefits from one session of deep and slow breathing on vagal tone and anxiety in young and older adults. *Sci. Rep.* **11**, 19267 (2021). 34588511 10.1038/s41598-021-98736-9 PMC8481564

[42] Jerath, R., Edry, J. W., Barnes, V. A. & Jerath, V. Physiology of long pranayamic breathing: neural respiratory elements may provide a mechanism that explains how slow deep breathing shifts the autonomic nervous system. *Med. Hypotheses* **67**, 566–571 (2006). 16624497 10.1016/j.mehy.2006.02.042

[43] Jolly, S. & Asokan, G. Mental training in general surgery: a qualitative review of Australian trainee perceptions. *ANZ J. Surg.* **94**, 63–67 (2024). 37485780 10.1111/ans.18620

[44] Spoon, D. B., Vickers, K. S. & Alkhouli, M. Mental skills training in cardiology. *J. Am. Coll. Cardiol.* **76**, 1905–1909 (2020). 33059836 10.1016/j.jacc.2020.09.530

[45] Tolin, D. F., O’Bryan, E. M., Davies, C. D., Diefenbach, G. J. & Johannesen, J. Central and peripheral nervous system responses to chronic and paced hyperventilation in anxious and healthy subjects. *Biol. Psychol.* **176**, 108472 (2023). 36481266 10.1016/j.biopsycho.2022.108472 PMC9839632

[46] Jansen, A. S., Nguyen, X. V., Karpitskiy, V., Mettenleiter, T. C. & Loewy, A. D. Central command neurons of the sympathetic nervous system: basis of the fight-or-flight response. *Science* **270**, 644–646 (1995). 7570024 10.1126/science.270.5236.644

[47] Pichardo, G. *What to know about 4-7-8 breathing*, https://www.webmd.com/balance/what-to-know-4-7-8-breathing (2025).

[48] Taraporewalla, K., Barach, P. & van Zundert, A. Teaching medical procedural skills for performance. *Clin. Pr.* **14**, 862–869 (2024). 10.3390/clinpract14030067 PMC11130924 38804399

[49] Snelgrove, H. & Gabbott, B. Critical analysis of evidence about the impacts on surgical teams of ‘mental practice’ in systematic reviews: a systematic rapid evidence assessment (SREA). *BMC Med. Educ.* **20**, 221 (2020). 32664909 10.1186/s12909-020-02131-3 PMC7362567

[50] Arora, S. et al. Development and validation of mental practice as a training strategy for laparoscopic surgery. *Surg. Endosc.* **24**, 179–187 (2010). 19633892 10.1007/s00464-009-0624-y

[51] Davison, S., Raison, N., Khan, M. S., Dasgupta, P. & Ahmed, K. Mental training in surgical education: a systematic review. *ANZ J. Surg.* **87**, 873–878 (2017). 28851014 10.1111/ans.14140

[52] Rao, A., Tait, I. & Alijani, A. Systematic review and meta-analysis of the role of mental training in the acquisition of technical skills in surgery. *Am. J. Surg.* **210**, 545–553 (2015). 26092443 10.1016/j.amjsurg.2015.01.028

[53] Anton, N. E. et al. Effects of a novel mental skills curriculum on surgical novices’ attention. *J. Surg. Res.* **219**, 86–91 (2017). 29078915 10.1016/j.jss.2017.05.112

[54] Lin, J. C., Paul, A. A. & Greenberg, P. B. Mental skills training and resident surgical outcomes: a systematic review. *J. Surg. Educ.* **77**, 1377–1391 (2020). 32773335 10.1016/j.jsurg.2020.05.028

[55] Vordenberg, S. E. et al. An integrative review of micro-credentials and digital badges for pharmacy educators. *Am. J. Pharm. Educ.* **88**, 100660 (2024). 38272238 10.1016/j.ajpe.2024.100660

[56] DeMarco, B., Ebanks, Y. & Tafuto, B. Digital badges in academia: an educational tool for the clinical research coordinator. *J. Clin. Transl. Sci.* **8**, e51 (2024). 38544747 10.1017/cts.2024.490 PMC10966829

[57] Wang, C., Bakhet, M., Roberts, D., Gnani, S. & El-Osta, A. The efficacy of microlearning in improving self-care capability: a systematic review of the literature. *Public Health* **186**, 286–296 (2020). 32882481 10.1016/j.puhe.2020.07.007

[58] Shay, A. Micro-credentialing: an option for clinical nurse specialists?. *Clin. Nurse Spec.* **37**, 299–300 (2023). 37870516 10.1097/NUR.0000000000000780

[59] Bideau, Y. M. & Kearns, T. A European approach to micro-credentials for lifelong learning and employability. *J. Eur. CME* **11**, 2147288 (2022). 36420424 10.1080/21614083.2022.2147288 PMC9677998

[60] Mashford-Pringle, A., Tan, S., Stutz, S. & Tjong, G. Designing accountability measures for health professionals: results from a community-based micro-credential: case study on indigenous cultural safety. *BMC Public Health* **23**, 879 (2023). 37173719 10.1186/s12889-023-15721-9 PMC10176280

[61] Desmarchelier, R. & Cary, L. J. Toward just and equitable micro-credentials: an Australian perspective. *Int. J. Educ. Technol. High. Educ.* **19**, 25 (2022). 35669714 10.1186/s41239-022-00332-y PMC9155197

[62] Kumar, J. A., Richard, R. J., Osman, S. & Lowrence, K. Micro-credentials in leveraging emergency remote teaching: the relationship between novice users’ insights and identity in Malaysia. *Int J. Educ. Technol. High. Educ.* **19**, 18 (2022). 35382441 10.1186/s41239-022-00323-z PMC8970641

[63] Mathur, A., Wood, M. E. & Cano, A. Mastery of transferrable skills by doctoral scholars: visualization using digital micro-credentialing. *Change* **50**, 38–45 (2018). 31656316 10.1080/00091383.2018.1510261 PMC6813843

[64] Peisachovich, E. H. et al. Using simulation-based methods to support demonstration of competencies required by micro-credential courses. *Cureus* **13**, e16908 (2021). 34513481 10.7759/cureus.16908 PMC8418224

[65] Clausen, J. M. Learning to fly: development and design of a micro-credentialing system for an educator preparation program in the absence of a required educational technology course. *TechTrends* **66**, 276–286 (2022). 34664042 10.1007/s11528-021-00673-x PMC8514802

[66] Gauthier, T. The value of microcredentials: the employer’s perspective. * J. Competency Based Educ.* **5**, e01209 (2020).

[67] Ericsson, K. A., Nandagopal, K. & Roring, R. W. Toward a science of exceptional achievement: attaining superior performance through deliberate practice. *Ann. N. Y. Acad. Sci.* **1172**, 199–217 (2009). 19743555 10.1196/annals.1393.001

[68] Mena-Guacas, A. F., Chacón, M. F., Munar, A. P., Ospina, M. & Agudelo, M. Evolution of teaching in short-term courses: a systematic review. *Heliyon* **9**, e16933 (2023). 37332952 10.1016/j.heliyon.2023.e16933 PMC10275963

[69] Starcke, K. & Brand, M. Decision making under stress: a selective review. *Neurosci. Biobehav. Rev.* **36**, 1228–1248 (2012). 22342781 10.1016/j.neubiorev.2012.02.003

[70] *National Microcredentials Framework*, https://www.education.gov.au/higher-education-publications/resources/national-microcredentials-framework (2025).

[71] Yeung, J. et al. Spaced learning versus massed learning in resuscitation - a systematic review. *Resuscitation* **156**, 61–71 (2020). 32926969 10.1016/j.resuscitation.2020.08.132

[72] Antoine, J., Dunn, B., McLanders, M., Jardine, L. & Liley, H. Approaches to neonatal intubation training: a scoping review. *Resusc Plus* **20**, 100776 (2024). 39376638 10.1016/j.resplu.2024.100776 PMC11456915

