# Supplementary material for: Neonatal endotracheal intubation: development of a comprehensive educational program for procedural skills
Source: Pediatr Res. 2025 Jun 17;99(2):484–9. doi: 10.1038/s41390-025-04217-4 (PMC12956573; doi:10.1038/s41390-025-04217-4)

Supplementary Material

Suppl. Nō	Document	Page Nō
S1	Literature search strategies for PubMed, Embase, Cochrane, Emcare, and CINAHL	2-4
S2	Criteria for Australian National Framework for Micro-credentials	5
S3	Instruction card	6-7
S4	Performance Checklist	8
S5	How to Practice 4-7-8 Breathing	9-10
S6	Mental skills training script	11-13
S7	Endotracheal intubation – Microcredential program outline	14
S8	A quality improvement activity protocol for the implementation of endotracheal-intubation micro-credential	15-17
Suppl. Figure S1	Flow diagram of study selection process	18

Search Strategy

Medline Via PubMed [29 January 2025]

Search Terms	Yield
(((Infant OR Neonate OR Neonat*) OR ("Infant"[Mesh] OR "Infant, Extremely Premature"[Mesh] OR "Infant, Extremely Low Birth Weight"[Mesh] OR "Infant, Very Low Birth Weight"[Mesh] OR "Infant, Small for Gestational Age"[Mesh] OR "Infant, Premature, Diseases"[Mesh] OR "Infant, Premature"[Mesh] OR "Infant, Postmature"[Mesh] OR "Infant, Newborn, Diseases"[Mesh] OR "Infant, Newborn"[Mesh] OR "Infant, Low Birth Weight"[Mesh] OR "Intensive Care, Neonatal"[Mesh])) AND (("Anesthesia, Endotracheal"[Mesh] OR "Intubation, Intratracheal"[Mesh]) OR (Tracheal Intubation OR Endotracheal Intubation))) AND (((simulation) OR ("Patient Simulation"[Mesh] OR "High Fidelity Simulation Training"[Mesh] OR "Simulation Training"[Mesh])) OR ((((((Coach) OR (Learning)) OR (Teaching)) OR (Coaching)) OR ("Teaching"[Mesh] OR "Education"[Mesh])) OR ("Mentoring"[Mesh])))	**746**

Embase (29 January 2025)

Search term	Yield
#1 infant/ or Infant.mp.	871326
#2 Neonate.mp. or newborn/	643088
#3 Extremely premature infant.mp. or extremely premature birth/	1008
#4 Preterm infant.mp. or prematurity/	140395
#5 low birth weight.mp. or low birth weight/	73419
#6 Very low birth weight.mp. or very low birth weight/	17996
#7 extremely low birth weight.mp. or low birth weight/ or extremely low birth weight/ or newborn/ or prematurity/ or very low birth weight/	732058
#8 Endotracheal intubation.mp. or endotracheal intubation/	70402
#9 tracheal intubation.mp. or endotracheal intubation/	69424
#10 nasotracheal intubation.mp. or nasotracheal intubation/	3239
#11 mentoring/ or Coaching.mp. or education/	522117
#12 Training.mp. or in service training/ or high fidelity simulation training/ or staff training/ or training/ or simulation training/	938480
#13 Tracheal tube.mp. or endotracheal tube/	28417
#14 1 or 2 or 3 or 4 or 5 or 6 or 7	1343300
#15 8 or 9 or 10 or 13	93966
#16 11 or 12	1360544
**#17 14 and 15 and 16**	**388**

Cochrane CENTRAL

Date Run: 29/01/2025 07:19:05

ID	Search	Hits
#1	Infant	67984
#2	Neonate	2873
#3	Preterm	19146
#4	Premature	24779
#5	Newborn	36983
#6	#1 OR #2 OR #3 OR #4 OR #5	93022
#7	Intubation	25998
#8	Endotracheal intubation	10741
#9	Tracheal intubation	7376
#10	#7 OR #8 OR #9	25998
#11	Teaching	25001
#12	Learning	42874
#13	Coaching	5952
#14	Mentoring	1598
#15	Education	118737
#16	Supervision	9091
#17	#11 OR #12 OR #13 OR #14 OR #15 OR #16	172755
#18	#6 AND #10 AND #17	**253**

Emcare 1995 to 2025 Week 3

#	Searches	Results
1	infant/ or Infant.mp.	236967
2	Neonate.mp. or newborn/	125019
3	Extremely premature infant.mp. or extremely premature birth/	415
4	Preterm infant.mp. or prematurity/	53515
5	low birth weight.mp. or low birth weight/	26439
6	Very low birth weight.mp. or very low birth weight/	7469
7	extremely low birth weight.mp. or low birth weight/ or extremely low birth weight/ or newborn/ or prematurity/ or very low birth weight/	168716
8	Endotracheal intubation.mp. or endotracheal intubation/	31655
9	tracheal intubation.mp. or endotracheal intubation/	31525
10	nasotracheal intubation.mp. or nasotracheal intubation/	1560
11	mentoring/ or Coaching.mp. or education/	233182
12	Training.mp. or in service training/ or high fidelity simulation training/ or staff training/ or training/ or simulation training/	391952
13	Tracheal tube.mp. or endotracheal tube/	14028
14	1 or 2 or 3 or 4 or 5 or 6 or 7	329977
15	8 or 9 or 10 or 13	41885
16	11 or 12	587170
17	14 and 15 and 16	**167**

CINAHL [29 January 2025]

**Search ID**	**Search terms**	Yield
S1	"Infant OR Neonate OR Newborn" OR (MH "Infant, Postmature") OR (MH "Infant, Premature") OR (MH "Infant+") OR (MH "Infant, Newborn+") OR (MH "Infant, Newborn, Diseases+") OR (MH "Infant, Hospitalized") OR (MH "Infant, Very Low Birth Weight")	313112
S2	MH "Intubation, Intratracheal+") OR (MH "Intubation+") OR (MH "Rapid Sequence Induction and Intubation") OR (MH "Endotracheal Tubes") OR "Endotracheal intubation OR Tracheal intubation"	22070
S3	S1 AND S2	2857
S4	(MH "Simulations+") OR (MH "Teaching+") OR (MH "Patient Simulation") OR (MH "Teaching Methods+") OR "Coaching OR Training OR Mentoring OR Learning OR Teaching OR Simulation" OR (MH "Learning Methods+") OR (MH "Teaching Methods, Clinical+") OR (MH "Teaching Materials, Clinical")	293275
**S5**	**S3 AND S4**	**147**

Total: 1701; Duplicates: 439; After removing duplicates: 1262

Criteria for Australian National Framework for Micro-credentials

1. Define the learning objectives: (1) The operator should be able to use mental imagery (eyes closed) to simulate the correct way of intubating infants while describing the steps out loud to a colleague, including points at which they would check and regulate their breathing rate [e.g., "just before commencing, I'm checking my respiration rate and am consciously slowing it down now", or "just before commencing, I'm checking my respiration rate and it is slow and calm, so I will now proceed."], (2) The operator should be able to demonstrate the correct way of intubating preterm and term mannequins independently (3) The operator should be aware of important points to monitor and manage their acute stress response, such as just prior to commencing the procedure and at any point during the procedure when they encounter a challenge [e.g., "my first attempt at ETI was unsuccessful, so I'm preparing for a second attempt. I've just monitored my breathing and am maintaining a slow, calm, regular rate. I'm now ready to proceed."].

2. Define the assessment criteria: (1) Operator should be able to perform all steps (n=11) in the "*Performance checklist – Endotracheal intubation*" "*correctly and independently*". (2) The time taken to obtain an adequate view should be <30 seconds. (3) The operator should be able to perform the slow breathing strategy correctly and have first simulated ETI using the technique of mental rehearsal.

3. Define the duration, content, and delivery method: *Duration:* The total duration of the program is 65 minutes. *Content:* The program includes (a) Watching instructional videos (25 min) (b) Reviewing instruction card (5 min) (c) Practice on term and preterm mannequins (10 min each = 20 min) as often as desired (d) *Assessment and feedback* (15 min) (e) *Delivery:* The program will need to be delivered in person in the NICUs. Any medical or nursing staff who wants to learn or refresh their intubation skills can take it. Any person proficient in intubation will be allowed to join as a coach and an assessor.

4. Create the content and assessment: The content and the approach to assessment that involves instructional videos

(https://www.youtube.com/watch?v=TgFFF3WK3LU, https://www.youtube.com/watch?v=IhRfQCG2i_o), MCQs, instruction card, and the checklist for prospective assessment (Figures 1-4 in supplementary file).

5. Pilot the micro-credential: The micro-credential should be piloted to ensure feasibility.

6. Launch the micro-credential: After successful piloting, the credential should be launched within the department/organisation.

7. Evaluate the effectiveness of micro-credential: Suggested methods to evaluate the effectiveness of the micro-credential is through prospective data collection using logbooks: (1) Frequency of program usage by staff and their satisfaction with it. (2) Number of intubation attempts, failed attempts, first attempt intubation success and time to successful intubation in practice sessions with mannequins and in term or preterm infants in clinical scenarios before and after implementation of the program. (3) Assessment of state anxiety while completing ETI using the PROMIS anxiety scale, (4) assessment of self-efficacy applying ETI using the PROMIS self-efficacy scale (i.e., confidence in applying ETI and managing challenges), (5) perceived stress of participants before vs after successful completion of the program using the PROMIS perceived stress scale, (6) satisfaction with the program using the ETI JITT Participant Satisfaction Survey. In addition. The ETI JITT Mental Rehearsal Monitoring Form can be used to track impacts of each mental rehearsal completed during the training.

Instruction Card

Endotracheal Intubation

Core Principles

Preparation: Laryngoscope and tube, **Basic check:** Suction, oxygen, warmer

Execution: **Position, Position, Position, Pressure, View, Insert, Check and Strap**

Behaviour: Professionalism, situational awareness, control acute stress response

Procedure

**Preparation**	Check intubation equipment, suction, oxygen, warmer
Operator **Position**	Bed height adjusted for optimal position of the operator – Baby's head levels with operator's upper abdomen or lower chest.
Baby's **Position**	• Baby's neck and thorax are in the midline, with both shoulders in a neutral position. Baby in sniffing position (Avoids neck hyperextension or hyperflexion).<br>• Stabilise the baby, as much as possible, prior to intubating.<br>• Consider premedication for non-emergency intubations.
Blade **Position**	Hold the laryngoscope in the left hand and introduce the blade in the oral cavity without hurting the baby's gums by opening the mouth with the right index finger and *advancing the tip of the laryngoscope blade step-by-step in the vallecula* (not too far in or out).
**View**	• Lift the entire laryngoscope in the direction of the handle to expose the glottis. May need to tilt the tip of the blade very slightly to lift the epiglottis.<br>• Apply cricoid pressure if required<br>• Suction, only if secretions are blocking the view

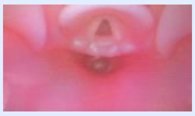	Optimum view – No corrective action required.
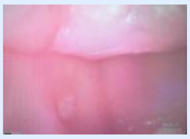	You see tongue/posterior pharynx *(**Reason: Blade not inserted far enough**)*. Corrective action: Advance until vallecula reached.
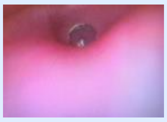	You see the oesophagus (***Reason: Blade too far in***). Corrective action: Withdraw slowly until the glottis appears.
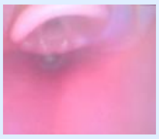	You see only part of the glottis *(**Reason: Blade off to one side**)*. Corrective action: Bring the blade in midline.
**Insertion**	• Hold and insert the ETT from the right side of the baby's mouth, maintaining a view of the vocal cord.<br>• Do not insert through the curved channel under the blade, as that will obstruct your view of the vocal cords.<br>• Do not force through closed vocal cords.
**Checking**	Check position and depth of insertion: Centimetre marking on ETT, Pedicap®, chest movement, breath sounds
**Strapping**	Hold the ETT in place by pressing it against the hard palate with your index finger while strapping.

In case of failed intubation attempt

	• Maintain professional demeanour.<br>• Control acute stress response.<br>• Maintain situational awareness.<br>• Attempt to stabilize the baby by effective bag-mask ventilation.<br>• Reconsider options – Retry, introducer, video laryngoscope, call for help, ventilate with mask, LMA

Performance Checklist – Endotracheal Intubation

Name of performer: **Designation:**

Name of assessor: **Date:**

A. Preparation

	Yes	No
Watched intubation video	☐	☐
Read the instruction card	☐	☐
Number of unsupervised practice sessions on the mannequin		
Number of supervised practice sessions on the mannequin		

B. Execution

Steps	Correct and independent	Incorrect or needed help
1. Checks Intubation Equipment	☐	☐
2. Ensures optimum position as the operator	☐	☐
3. Ensures the optimum position of the baby	☐	☐
4. Considers the need for premedication	☐	☐
5. Administers PPV using a mask before intubation	☐	☐
6. Correctly introduces laryngoscope blade in baby's mouth	☐	☐
7. Obtains an adequate view	☐	☐
• **Time to obtain an adequate view in seconds**		
8. Does not use the blade's curved space for passing the ETT	☐	☐
9. Inserts ETT through vocal cords	☐	☐
10. Confirms ETT position	☐	☐
11. Checks ETT depth and corrects it if needed	☐	☐
12. Holds ETT securely by pressing against the hard palate	☐	☐
13. Assists ETT strapping at an appropriate depth	☐	☐

How to Practice 4-7-8 Breathing

The body prepares us to deal with a perceived threat by activating the sympathetic nervous system (fight, flight, freeze response). Adrenaline is released, respiration rate (hyperventilation) and heart rate increase to mobilise oxygen, sweating cools the body, and so on.

When we are not actually fighting or fleeing from the perceived threat (e.g., because there is not an objective threat, or it is inappropriate to fight or flee), hyperventilation causes blood carbon dioxide levels to drop, leading to respiratory alkalosis (increased blood pH). Respiratory alkalosis leads to a range of uncomfortable physical symptoms such as light-headedness, dizziness, dry mouth, tingling extremities, and perceived shortness of breath. Despite the feeling of shortness of breath, the opposite is actually true – blood becomes too alkaline, which can only be corrected by reducing oxygen intake or increasing carbon dioxide levels. The symptoms of alkalosis make it very difficult to concentrate on the task at hand, which is a problem when you are responsible for administering a medical procedure like endotracheal intubation.

Sometimes people hyperventilate in response to an acute stressor (e.g., unexpected need to deliver a time-sensitive and critical procedure), which leads to rapid alkalosis, whereas other people chronically hyperventilate (i.e., elevated respiration gradually increases alkalosis over time, leaving them more vulnerable if they encounter a daily stressor).
To re-establish equilibrium, you need to slow down your breathing rate. This is a very practical and simple technique that you can use anywhere at any time.

You can try the *“4-7-8 breathing*”, which will restore the balance of oxygen and carbon dioxide in your blood. The technique can be practised anywhere and at any time and as often as desired. You might want to start with four cycles in a row, and then work up to eight cycles. You may feel a bit lightheaded at first, but it will pass. The steps of the 4-7-8 breathing technique are:

1. Find a comfortable place to sit with your back straight.
2. Place your tongue against the back of your top teeth and keep it there.
3. Exhale completely through your mouth around your tongue, making a whoosh sound. Purse your lips if it helps.
4. Close your lips and inhale through your nose for a count of four.
5. Hold your breath for a count of seven.
6. Exhale completely through your mouth, making a whoosh sound for a count of eight.
7. This completes one cycle. Repeat for three more cycles.

The 4-7-8 breathing can be practised anytime when the participants feel stressed to reduce acute hyperventilation or routinely to reduce chronic hyperventilation.

If you don't remember this procedure in detail when you feel stressed, **the most important thing to remember is to *slow your breathing rate down***. This will restore your blood pH to equilibrium, reduce the intensity of any uncomfortable physical sensations, and also anchor your attention in the present moment rather than the past or future. Focusing as much of your attention as possible on the present moment will be helpful for task performance.

This technique is always available when and where you need it. Two critical times to remember to monitor and manage your respiration rate are (1) just prior to commencing ETI and (2) during the procedure if you encounter any challenges (e.g., difficulties with the procedure, managing a distressed family member).

What to Know About 4-7-8 Breathing. WebMD June 27, 2023
https://www.webmd.com/balance/what-to-know-4-7-8-breathing
Accessed 9th July 2024

Mental Skills Training Script

Mental Skills Training (MST) is a form of cognitive simulation to aid technical skill development, whereas preoperative mental rehearsal is used for preparation, often immediately before performing the practical skill. The method of MST in our program involves internal monologue (self-talk based on the MST script) while mentally going through the steps of intubation anywhere and anytime, including just before the practice session on mannequins and intubation in a clinical set-up.

In addition to simulating the steps of intubation with an internal monologue, MST in this program will incorporate awareness of a range of contextual cues using all your senses (visual, auditory, olfactory, tactile) and reflections on your emotional experience. Within the mental simulations, you will practice noticing these contextual cues (e.g., sounds, other people around, reassuring parents and asking them to leave the room) and emotional (e.g., noticing stress symptoms and practising managing them) cues and responding to them in ways that will achieve optimal performance on the ETI. You will also mentally simulate completing ETIs that do not go smoothly so that you can 'practice' corrective procedures while managing your emotional response. You will include slow breathing in your mental simulations, just as you will in your practice simulations with the mannequins and when administering ETI with real patients thereafter.

Below are two scripts that could be used to guide mental simulations. Script 1 outlines a standard ETI procedure that goes smoothly. Script 2 is an example of a script that incorporates more contextual information and that presents more challenges.

Script 1. ETI Procedure

Close your eyes or avert your gaze to the ground. See yourself with an infant you are about to intubate. See the infant in your mind's eye now.

Check that your breathing is slow and regular. Make sure that as much of your attention as possible is on the task at hand in the present moment. How are you feeling in your body? How are you feeling emotionally? Continue to breathe slowly and focus your attention on the present until you feel calm and focused on the task at hand.

Imagine yourself checking the equipment—what exactly are you checking? What do you need? See everything you need in front of you.

See yourself now getting into the optimum position and ensuring the baby, too, is in the optimum position.

Now, see yourself picking up the laryngoscope and introducing the blade into the baby's mouth.

See yourself getting an adequate view and inserting the ETT through the vocal cords without using the blade's curved space for passing the ETT.

See yourself confirming the position, checking the depth, and correcting the position as needed.

Finally, imagine yourself holding the ETT securely by pressing against the hard palate and assisting with strapping it at an appropriate depth. Notice how it feels to have completed the procedure successfully, and consider what this means about your ability to do it in the future.

Script 2. Procedure with Contextual Cues

Close your eyes or avert your gaze to the ground.

Imagine arriving at work tomorrow morning. The beeping equipment, disinfectant, and fluorescent lighting are all familiar. How are you feeling, and where are you feeling it in your body? What other sounds can you hear? What else can you see around you?

You see a nurse rushing up to you, and she says you are needed urgently to intubate an infant. You haven't had to intubate an infant for a while, so you initially hesitate, and you can feel your heart rate increasing a little. You focus back on the task and hand and recall your consultant's calm voice talking you through the procedure at training. You can hear her voice calmly guiding you through the steps. You remember you've practised this plenty of times. You note that your breathing rate has increased, so you intentionally slow it down and immediately start to feel calmer.

You focus all your attention on the present moment and kick into action. You recall the first step of checking the intubation equipment – see yourself checking the equipment now. Now, see yourself ensuring that you and the baby are in a good position. What is the baby doing? Is the baby animated or limp? You catch one of the parent's eyes, and she looks terrified and cries. You give her a brief nod, reassure her it will be ok, you and the nurses are looking after her baby, you calmly ask a nurse to take the mother out of the room, and then shift your attention immediately back to the task at hand. You introduce the laryngoscope blade into the baby's mouth as you have been trained to do; you remember not to use the blade's curved space for passing the ETT. See yourself placing the laryngoscope blade into the baby's mouth. You are feeling focused and calm. You remember to slow your breathing down and gently shift your attention back to the task at hand.

You make sure you have an adequate view and then insert the ETT through the vocal cords, confirm the position, check the depth and notice it is a bit out of place, so you correct it. You hold the ETT securely by pressing against the hard palate and assist the ETT strapping at an appropriate depth. Your role is complete, and you hand it over to the nursing staff. Notice the feeling of satisfaction you feel and savour that feeling for a moment. At that moment, you know you can do it again in the future when needed. You again reassure the baby's mother and then head off to start your rounds.

It is important for you to write and record your own scripts with more details about aspects of the procedure you are least confident about. You should record scripts where the procedure does not go smoothly so that you can mentally rehearse corrective actions. For example, you might have scripts describing:

- Having difficulty exposing the glottis (only see tongue/posterior pharynx)
- You only see part of the glottis
- You see the oesophagus
- Having difficulty exposing the vocal cords
- Nursing/medical staff or family responding in helpful/unhelpful ways
- Need to stabilise the baby by effective bag-mask ventilation
- Need to retry a second or third time

You can then listen to these scripts while 'seeing' yourself experience them within mental imagery. Your mental imagery may be vivid or fuzzy. Around 2% of the population are aphantasic and cannot see mental images at all. The most important thing is that you attempt to connect with as many visual, physical, auditory, and emotional cues as possible as you are listening to the script and talking yourself through the procedure.

Procedural skills in neonatal endotracheal intubation

The content, duration, and delivery of the microcredential

Step 1: Preparation (60 min online)

(a) Watch the instructional videos* including one on deep breathing technique* (50 min)

(b) Review instruction card and mental skills training script (10 min)

Step 2: Face-to-face coaching session (30 min)

(c) Coach demonstrates the technique and corrective measures step-by-step (15 min).

(d) Trainee performs the procedure in the same sequence, describing each step clearly (15 min).

Step 3: Practice on mannequins (Supervised/Unsupervised) (Time variable: It will be determined by the time the trainee takes to acquire skills)

(e) Trainee practices on term and preterm mannequins (10 min each) as often as desired,

Step 4: Assessment (15 min)

(f) Assessment using the performance checklist and feedback.

Total duration: 125 min

Delivery:

The program is delivered in person in the NICU. Any medical or nursing staff member wanting to learn or refresh their neonatal endotracheal intubation skills can take it. Any NICU team member proficient in the procedure can participate as a coach and assessor.

***Instructonal videos:**

i. https://www.youtube.com/watch?v=TgFFF3WK3LU,

ii. https://www.youtube.com/watch?v=IhRfQCG2i_o

iii. Breathing to Activate Parasympathetic Nervous System (youtube.com)

A quality improvement activity protocol for the implementation of endotracheal-intubation micro-credential

Background

Procedures such as endotracheal intubation and chest drain insertion are critical and life-saving aspects of neonatal intensive care. However, acquiring and maintaining competency in these procedures is becoming difficult due to restricted working hours, an increased number of trainees, and, in the case of intubation, increasing preferential use of non-invasive ventilation. There was no standard educational and credentialing program focusing on the development and maintenance of neonatal endotracheal intubation skills.

We developed an evidence-based, comprehensive program to assist the development and, importantly, maintain the skills for ETI in neonates. The inclusion of Just-In-Time training (JITT), Rapid Cycle Deliberate Practice (RCDP), deep and slow breathing (DSB) and MST make it innovative, and the structure and delivery as micro-credentials are innovative aspects of this program. This quality improvement project aims to implement the program and assess its benefits. If found beneficial, the program has the potential to expand its scope to include other medical and nursing procedures, thereby enhancing the overall safety and quality of procedural training in neonatology.

Methods

The standard guidelines and tools will be used to undertake this QI activity (Weekes 2018, Clinical Excellence Commission, NSW).

Objectives:

1. Improve clinicians' confidence in performing the procedure (e.g., endotracheal intubation, chest drain insertion)
2. Increase success rate in the procedure (e.g., success in intubation on the first attempt, success in insertion of chest drain)
3. Reduce procedure-related adverse events (e.g., trauma, desaturations during intubation, cardiac arrest)

A. Eligible healthcare personnel: All medical and nursing staff involved in the procedure.

B. Logistic setup:

   1. A room close to the clinical area will be used for JITT. The room will have a desktop with an internet connection.

2. Term and preterm mannequins, mannequins for chest drainage, video laryngoscope, chest drains, and other consumables required for intubation and chest drain placement will be secured.
3. Videos of intubation and chest drain placement, laminated, easy-to-understand cards with instructions for the correct method of intubation and chest drain placement, and a RedCap database for documenting program usage and outcomes.
4. The program users will receive training from one of the JITT trainers. The JITT trainer will introduce users to the appropriate use of JITT resources, the correct technique of the procedures and RedCap documentation. After the introduction, the users will be granted access to the room.
5. The trainer will assess the program user with the help of a standard checklist and grant credentials if all the items are completed.

C. JITT training components: Procedure practice on mannequin aided by procedure video and instruction cards. Although not mandatory, it is advisable to have another person to supervise the practice and give suggestions.

D. Suggested time to perform JITT and the strategies to control acute stress response :

(1) Just before the procedure, if time allows (e.g., non-emergency intubation)

(2) As a part of the daily routine (e.g., start of the shift)

(3) As and when time allows, as per individual needs

JITT is not a compulsory but an optional training tool.

E. Monitoring: The data collection sheets have been attached. The following data will be collected:

1. Frequency of the use of JITT
2. User profile and satisfaction
3. Regular audits of procedural success rate and complications
4. Regular audits of the relevant clinical outcomes.

Future directions

To expand the scope of JITT to include more procedures, such as umbilical catheter placement and removal of the umbilical line, as well as strapping and adjusting the endotracheal tube.

References

1. Patocka C, Pandya A, Brennan E, et al. The impact of just-in-time simulation training for healthcare professionals on learning and performance outcomes: a systematic review. Sim Healthcare 2024;19:S32-S40.
2. Gizicki E, Assaad M-A, Masse E, et al. Just-In-Time neonatal endotracheal intubation simulation training: a randomized controlled trial. J Pediatr. 2023;261:113576.

3. Scholtz AK, Monachino AM, Nishisaki A, et al. Central venous catheter dress rehearsals: translating simulation training to patient care and outcomes. Simul Healthc. 2013 Oct;8(5):341-9.
4. Weekes L, Lawson T, Hill M. How to start a quality improvement project. BJA Educ. 2018 Apr;18(4):122-127.
5. https://www.cec.health.nsw.gov.au/CEC-Academy/quality-improvement-tools

Supplementary Figure S1: Flow diagram of study selection process

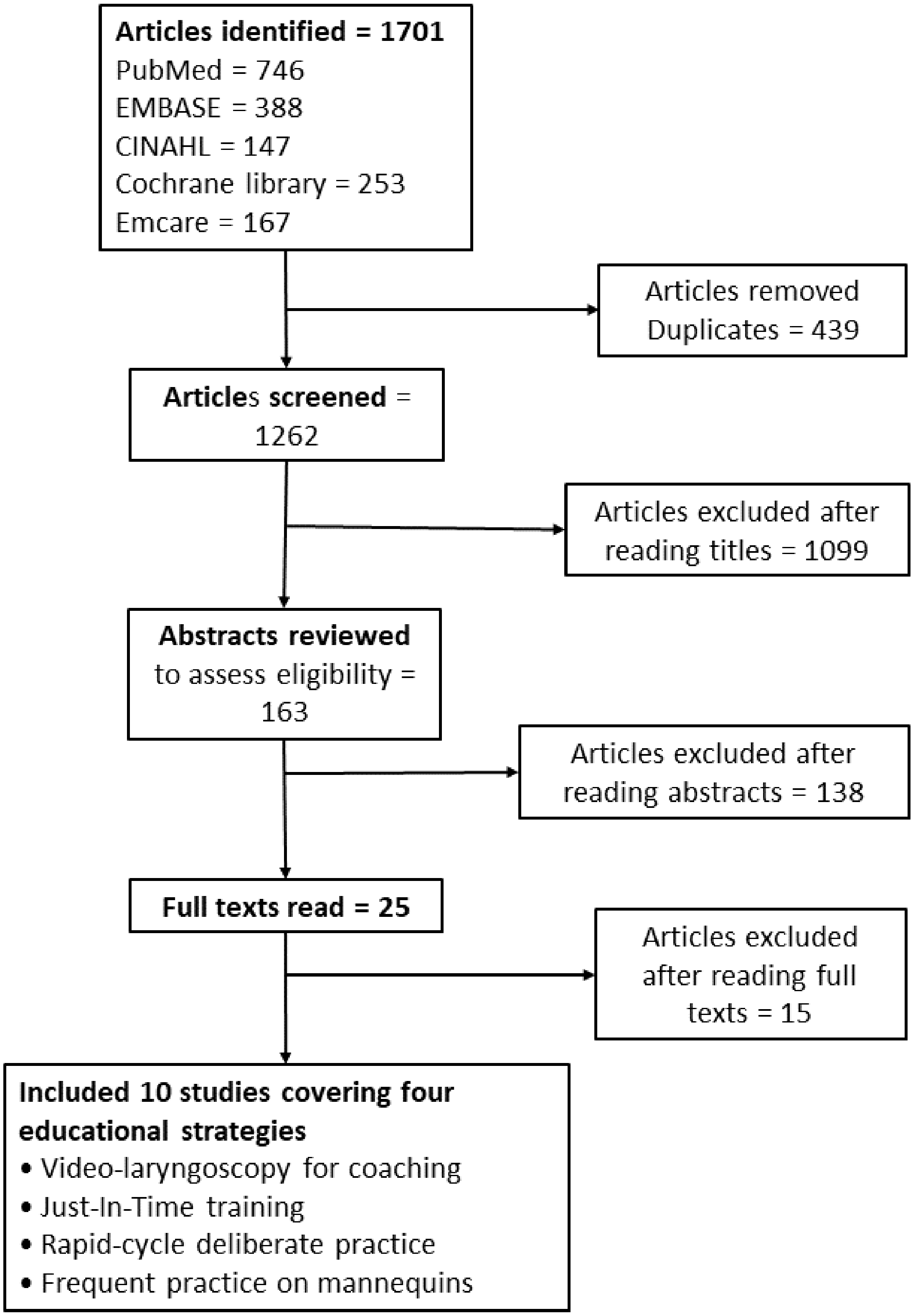

Supplement: Supplementary file 1 — Supplementary Information [file 41390_2025_4217_MOESM1_ESM.pdf]
